# Methamphetamine Increases LPS-Mediated Expression of IL-8, TNF-α and IL-1β in Human Macrophages through Common Signaling Pathways

**DOI:** 10.1371/journal.pone.0033822

**Published:** 2012-03-29

**Authors:** Xun Liu, Peter S. Silverstein, Vijeta Singh, Ankit Shah, Nilofer Qureshi, Anil Kumar

**Affiliations:** 1 Division of Pharmacology and Toxicology, School of Pharmacy, School of Medicine, University of Missouri-Kansas City, Kansas City, Missouri, United States of America; 2 Shock/Trauma Research Center, School of Medicine, University of Missouri-Kansas City, Kansas City, Missouri, United States of America; University of Nebraska Medical Center, United States of America

## Abstract

The use of methamphetamine (MA) has increased in recent years, and is a major health concern throughout the world. The use of MA has been associated with an increased risk of acquiring HIV-1, along with an increased probability of the acquisition of various sexually transmitted infections. In order to determine the potential effects of MA exposure in the context of an infectious agent, U937 macrophages were exposed to various combinations of MA and bacterial lipopolysaccharide (LPS). Treatment with MA alone caused significant increases in the levels of TNF-α, while treatment with both MA and LPS resulted in significant increases in TNF-α, IL-1β and the chemokine IL-8. The increases in cytokine or chemokine levels seen when cells were treated with both LPS and MA were generally greater than those increases observed when cells were treated with only LPS. Treatment with chemical inhibitors demonstrated that the signal transduction pathways including NF-kB, MAPK, and PI3-Akt were involved in mediating the increased inflammatory response. As discussed in the paper, these pathways appear to be utilized by both MA and LPS, in the induction of these inflammatory mediators. Since these pathways are involved in the induction of inflammation in response to other pathogens, this suggests that MA-exacerbated inflammation may be a common feature of infectious disease in MA abusers.

## Introduction

The abuse of methamphetamine (MA) is a major problem in many parts of the world, including the United States of America, Eastern Europe and Southeast Asia [1], [2]. A recent study estimated that over 10 million people, age 12 years and older, had tried MA at least once in their lives [3]. The chemical similarity between MA and the neurotransmitter dopamine appears to be the basis for many of the effects of this drug [4], [5].

Most studies on MA have focused on the effects of the drug in the CNS where it has been shown to interact with dopamine transporters (DAT) and dopamine receptors (D1-D5) (reviewed in [6]). In the CNS, much of the MA-induced toxicity can be related to changes in dopamine disposition as a result of altered expression and activity of DAT and vesicular monoamine transporter-2 [6], [7]. The neurotoxic effects of MA have also been shown to be mediated through dopamine receptors. Antagonists of D1 and D2 have been shown to ameliorate the neuroxic effects of MA in the CNS in animal models [8], [9].

In the peripheral immune system, MA or dopamine have been shown to affect peripheral blood mononuclear cells (PBMC), macrophages and dendritic cells [10], [11], [12], [13]. Exposure of mouse bone marrow-derived dendritic cells to MA was demonstrated to negatively impact antigen presentation and processing. MA caused alkalization of endosomes and lysomes, and blocked antigen presentation. Furthermore, treatment with MA inhibited phagocytosis by mouse bone marrow-derived macrophages [13]. Treatment of monocyte-derived dendritic cells with MA has been demonstrated to result in increased expression levels of the chemokine receptors CXCR4 and CCR5 [14]. Through the use of D1 and D2 antagonists, it was demonstrated that both of these dopamine receptors were involved in mediating the increase in the chemokine receptors. Treatment of human monocyte-derived macrophages with MA or dopamine was also shown to increase infection of these cells with HIV-1, as well as to increase viral replication; these effects were mediated by either D1 or D2 [10], [11]. Similar results regarding HIV-1 infectivity in monocyte-derived dendritic cells have also been reported [14]. Proteomic analyses of PBMC isolated from HIV+ donors demonstrated that MA treatment also altered the abundance of a number of proteins, including several involved in mediating the effects of oxidative stress. Compared to untreated PBMC, the levels of glutathione-S-transferase, superoxide dismutase and peroxiredoxin 6 were reduced in PBMC treated with MA [12]. Analysis of microarray data obtained from MA-treated monocyte-derived dendritic cells, followed by confirmation using real-time PCR, revealed that exposure to MA resulted in increased expression of TNF-α, IL-1β, and IL-8 [15].

In contrast to the effects of MA on macrophages, the molecular aspects of LPS interactions with macrophages have been extensively studied for more than 3 decades and numerous reviews have covered relevant signal transduction pathways in exquisite detail (reviewed in [16], [17], [18]). Briefly, LPS first interacts with LPS binding protein which promotes the subsequent interaction of LPS with CD14. LPS is then transferred to the TLR4/MD2 complex which causes TLR4 to oligimerize, and this results in the recruitment of TIR adaptor proteins (Mal, MyD88, TRIF, TRAM and SARM). TLR4 signaling is then mediated through the MyD88-dependent and MyD88-independent pathways, the former leading to the induction of inflammatory cytokines while the latter leads to the induction of Type I interferons. In the MyD88-dependent pathway, MyD88 recruits the kinase IL-1 receptor-associated kinase 4 (IRAK4). IRAK-4 then activates another kinase of the same family, IRAK 1. IRAK 1 interacts with TRAF6 and together they activate TGF-β–activated kinase 1 (TAK 1). TAK 1 then activates IKK of the NF-κB pathway, and TAK 1 also activates the MAPK pathway. IKK activation leads to the phosphorylation of inhibitor IκB which is then degraded by the proteasome. This allows translocation of active NF-κB to the nucleus where this transcription factor is involved in the production of pro-inflammatory cytokines and chemokines such as TNF-a, IL-8, and IL-1β. In contrast, the MyD88-independent pathway leads to the activation of IRF3 and the induction of Type I interferons. The MyD88-independent pathway also leads NF-κB activation, but this occurs later than the activation through the MyD88-dependent pathway.

Despite the prevalence of MA abuse, and the high coincidence of bacterial infections among MA abusers, few studies have attempted to determine the effects of MA on the production of inflammatory cytokines in macrophages. One study using THP-1 macrophages found that treatment with only MA produced no significant changes in IL-1β production, but MA did act to increase IL-1β production stimulated by LPS [19]. In another recent study using human monocyte-derived macrophages, the effects of LPS and MA on secretion of matrix metalloproteinase-9 (MMP-9) were monitored. Although MA alone had no effect on MMP-9 secretion, MA did significantly increase the level of MMP-9 secretion induced by LPS [20].

The clinical relevance of potential MA-LPS interactions led us to investigate the effects of chronic MA exposure on LPS-mediated induction of inflammatory cytokines. To address this question we utilized PMA-differentiated U-937 cells, as these are a well established model of human macrophages. These cells were treated for 48 h with MA and then exposed to LPS for three hours. Expression levels of inflammatory cytokines were determined at both the mRNA and protein levels. Furthermore, to enhance our understanding of MA-LPS interactions, the functions of several signaling pathways in mediating these interactions were determined.

## Materials and Methods

### Cell Culture and Treatment

The U937 monocytic cell line was obtained from ATCC (Manassas, VA) and maintained in Roswell Park Memorial Institute (RPMI) 1640 media (Sigma Aldrich, St. Louis, MO) containing 10% FBS. U937 monocytes were differentiated into macrophages by treatment with 100 nM phorbol 12-myristate 13-acetate (PMA) for 48 h. The lipopolysaccharide (ReLPS) was isolated from a deep rough mutant of Escherichia coli D31 m4 [21]. Cells were cultured with LPS (100 ng/ml), 500 µM methamphetamine or LPS and MA at the times indicated in the figure legends. In the experiments with inhibitors, the macrophages were cultured with either 20 µM SC-514 (IκB kinase inhibitor), 10 µM SB203580 (p38 MAPK inhibitor), 10 µM LY294002 (PI3K inhibitor) or 5 µM U-0126 (MEK1/2 inhibitor) (Cayman Chemicals, Ann Arbor, Michigan) 1 h prior to the addition of each dose of methamphetamine or LPS. After 3 h stimulation with LPS, cells were harvested for RNA isolation. In an additional experiment the cells were harvested after 24 h incubation with the various combinations of MA and LPS for the analysis of supernatant cytokines.

### Real time RT-PCR and cytokine protein assays

Real time reverse transcriptase polymerase chain reaction (RT-PCR) was used to determine the mRNA expression in a Bio-Rad iCycler. Total RNA was extracted using an RNeasy kit (Qiagen, Valencia, CA) as described in the manufacturer's protocol. The reaction conditions included reverse transcription at 50°C for 30 min, 95°C for 15 min and 45 cycles at 95°C for 15 sec and 62°C for 30 sec then at 74°C for 15 sec. Separate amplification of hypoxanthine-guanine phosphoribosyl-transferase (HPRT) was used to normalize gene expression. Fold-differences were calculated using the 2^−ΔΔCT^ method [22].

The PCR probes and primers used were as follows: IL-8 forward primer 5′ CTC TTG GCA GCC TTC CTG ATT 3′, reverse primer 5′ TAT GCA CTG ACA TCT AAG TTC TTT AGC A 3′, and probe 5′ FAM-CTT GGC AAA ACT GCA CCT TCA CAC AGA-3′ BHQ; IL-1β forward primer 5′ -ACA GAT GAA GTG CTC CTT CCA- 3′ and reverse primer 5′-GTC GGA GAT TCG TAG CTG GAT- 3′; TNF-α forward primer 5′ -CCC AGG GAC CTC TCT CTA ATC- 3′ and reverse primer 5′-ATG GGC TAC AGG CTT GTC ACT- 3′; HPRT forward primer 5′-GCT TTC CTT GGT CAG GCA GTA- 3′ and reverse primer 5′ -CCA ACA CTT CGT GGR GTC CTT T- 3′.

Cell culture supernatants were collected and the protein concentrations of cytokines were analyzed by the Bio-Plex multi-cytokine bead assay system (Life Science Research, Hercules, CA) according to the manufacturer's instructions. The protein expression was measured using Bio-Plex Manager 5.0 software and the fold changes were calculated by comparing the values with the 5PL standard curve.

### Western Blotting

U937 differentiated macrophages were harvested at the time points indicated and nuclear and cytoplasmic extracts were prepared using the NE-PER Nuclear extraction kit (Pierce, Rockford, IL). Briefly, 20 µg of protein was loaded on a 10% acrylamide gel and electrophoresed at 90 V for about 3 h and transferred to a PVDF membrane at 350 mA for 90 min. The expression of p50 was detected using NF-κB p50 (H-119) (1∶1500) primary antibody (Santa Cruz Biotechnology, Inc.). Lamin-B (C-20) (1∶1500) and GAPDH (FL-335) (1∶2000) were used as endogenous controls for the nuclear extracts and for the cytoplasmic extracts respectively. HRP-conjugated secondary antibodies were utilized to detect the primary antibodies and proteins were visualized by BM Chemiluminescence Western Blotting Substrate (POD) (Roche Applied Sciences, Indianapolis, IN). Quantification was performed by spot densitometry using FluorChem HD2 software (Alpha Innotech, San Leandro, CA).

## Results

### Methamphetamine augments the induction of inflammatory cytokines by LPS

**Figure 1:**
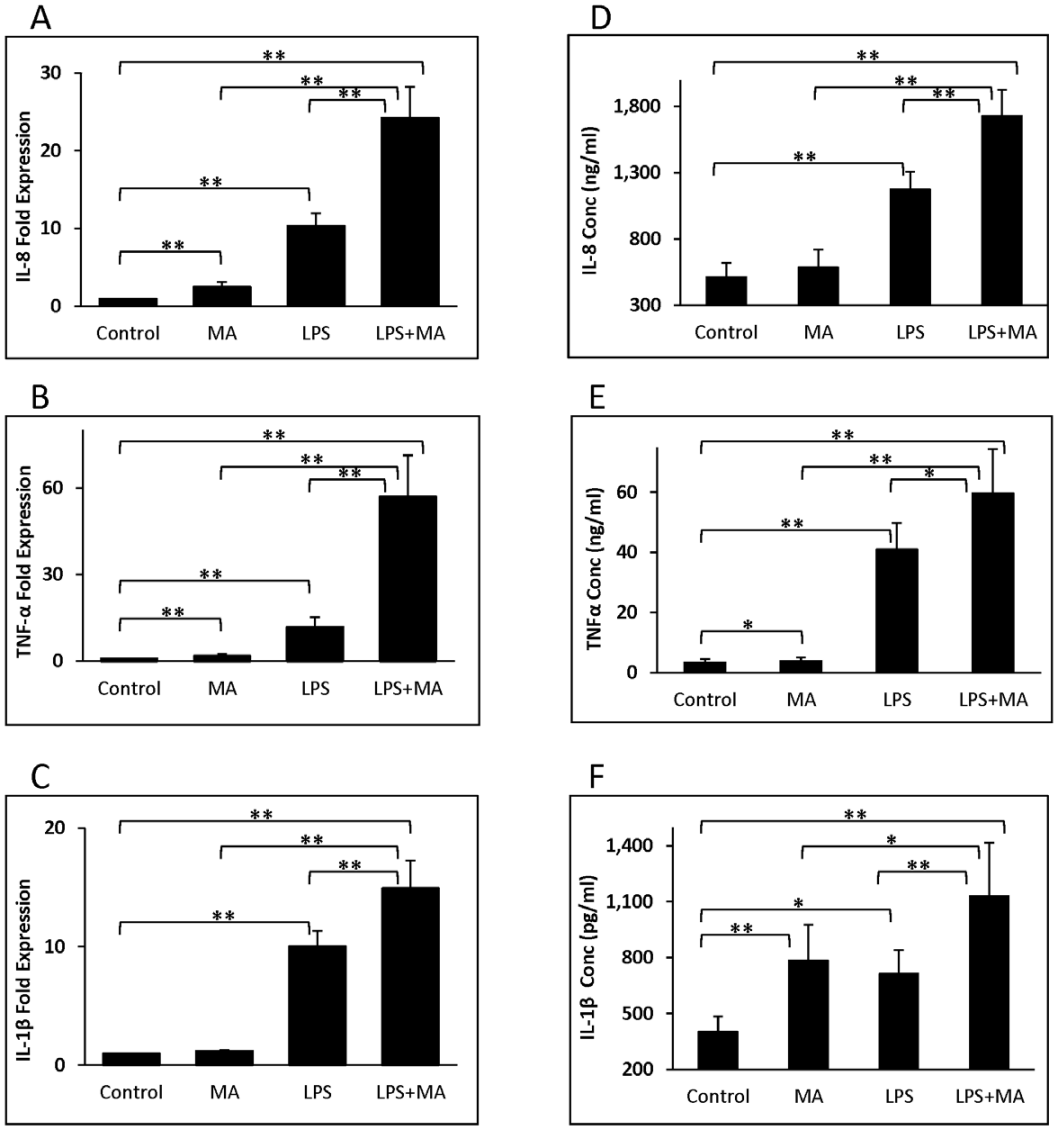
Treatment of U937 macrophages with MA or/and LPS induced an increase in IL-8, TNF-α, and IL-1β. Cells were differentiated into macrophages by treatment with 100 nM PMA for 48 h. Cells were treated with MA at 0, 24 and 48 h and then stimulated with 100 ng/ml LPS for 3 h, after which they were harvested. RNA was isolated and analyzed with real-time RT-PCR (A–C). In a separate experiment, cells were treated with MA at 0, 24 and 48 h then stimulated with 100 ng/ml LPS for 24 h and the cytokines in supernatants were measured using Bio-Plex (D–F). The error bars show standard error of three independent experiments. *: p<0.05; **: p<0.01.

U937-differentiated macrophages were treated with bacterial LPS and MA (individually as well as in combination) and then cytokine expression was quantitated at the levels of mRNA and protein. The concentration of MA (500 µM) was chosen because it is midway between 240 and 1144 µM, the concentration range calculated to be present in the spleen during binge use of the drug [13]. The calculation is based on reports of serum concentrations of MA and data regarding the serum/tissue ratio of MA in various organs [23]. Treatment of U937 macrophages with MA and LPS resulted in cytokine levels that were generally higher than those observed when cells were treated with LPS alone. For IL-8, treatment with MA resulted in IL-8 levels 2.5±0.2- fold higher than control and LPS treatment resulted in levels 10.4±0.5-fold higher than untreated controls at the RNA level, whereas treatment with LPS in conjunction with MA resulted in IL-8 levels that were 24.3±1.3-fold higher than controls (Figure 1A). For IL-8 expression at the protein level, MA treatment showed only slightly higher levels than the untreated control, while LPS produced significantly higher levels of IL-8 protein that were further increased by concomitant treatment with LPS and MA (Figure 1D). In the case of TNF-α, MA treatment resulted in RNA levels that were approximately twice those of untreated cells, LPS treatment resulted in levels 11.8±1.1- fold higher than controls, whereas treatment with LPS in conjunction with MA resulted in TNF-α levels that were 57±4.8-fold higher than untreated controls (Figure 1B). Increases in TNF-α at the protein level paralleled the increases seen at the RNA level, but to a lesser degree (17.6-fold difference in protein in cells treated with MA and LPS compared to control) (Figure 1E). For IL-1β, MA treatment resulted in RNA levels that were slightly higher than that of control, while LPS and LPS +MA treatments resulted in cytokine expression levels that 10.1±1.3 and 15.0±2.3- fold higher than controls, respectively (Figure 1C). For IL-1β expression at the protein level, MA and LPS produced an almost 2-fold increase in cytokine level when used individually and an increase of almost 3-fold when cells were treated with both agents at once (Figure 1F). For all three cytokines/chemokine, IL-8, TNF-α, and IL-1β, the levels of both RNA and protein induced by cotreatment with both LPS and MA were significantly higher than the levels induced by treatment with each agent alone.

### LPS and MA cause an increase in NF-κB p50 in the nucleus

**Figure 2:**
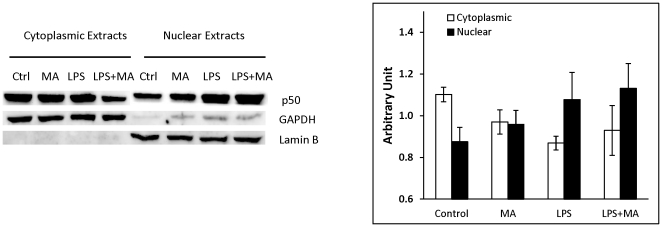
Treatment of U937 macrophages with MA ± LPS resulted in increased translocation of the p50 subunit of NF-κB. 1×10^6^ U937 monocytes were differentiated into macrophages with 100 nM PMA for 48 h. Cells were treated with MA at 0, 24 and 48 h then stimulated with 100 ng/ml LPS for 3 h. Cytosolic and nuclear protein fractions were purified by electrophoresis on 10% SDS gel and transfer to a PVDF membrane. The cytosolic fractions and nuclear fractions are shown in a representative western blot. The expression of the p50 subunit was normalized to the appropriate compartmental housekeeping genes (Lamin B for nucleus and GAPDH for cytoplasm). The means and SE values are from three independent experiments.

The involvement of the NF-κB pathway in the regulation of the expression of inflammatory cytokines has been well documented. In order to gain some insight into the effects of MA and LPS on NF-κB function, U937 macrophages were treated for 48 h with 500 µM MA, followed by exposure to LPS for 3 h. Cells were then processed for the isolation of nuclear and cytoplasmic protein fractions which were assayed for p50 expression by western blot analysis (Figure 2). As expected, treatment with LPS resulted in a significant increase in nuclear localization of p50 compared to that of control. However, an increase in the nuclear/cytoplasmic ratio of p50 for cells treated with MA alone was also observed. The nuclear/cytoplasmic ratio of p50 for cells treated with only LPS was not significantly different than the ratio for cells treated with MA and LPS.

### Cytokine induction mediated by a combination of LPS and MA is abrogated by an inhibitor of NF-κB

**Figure 3:**
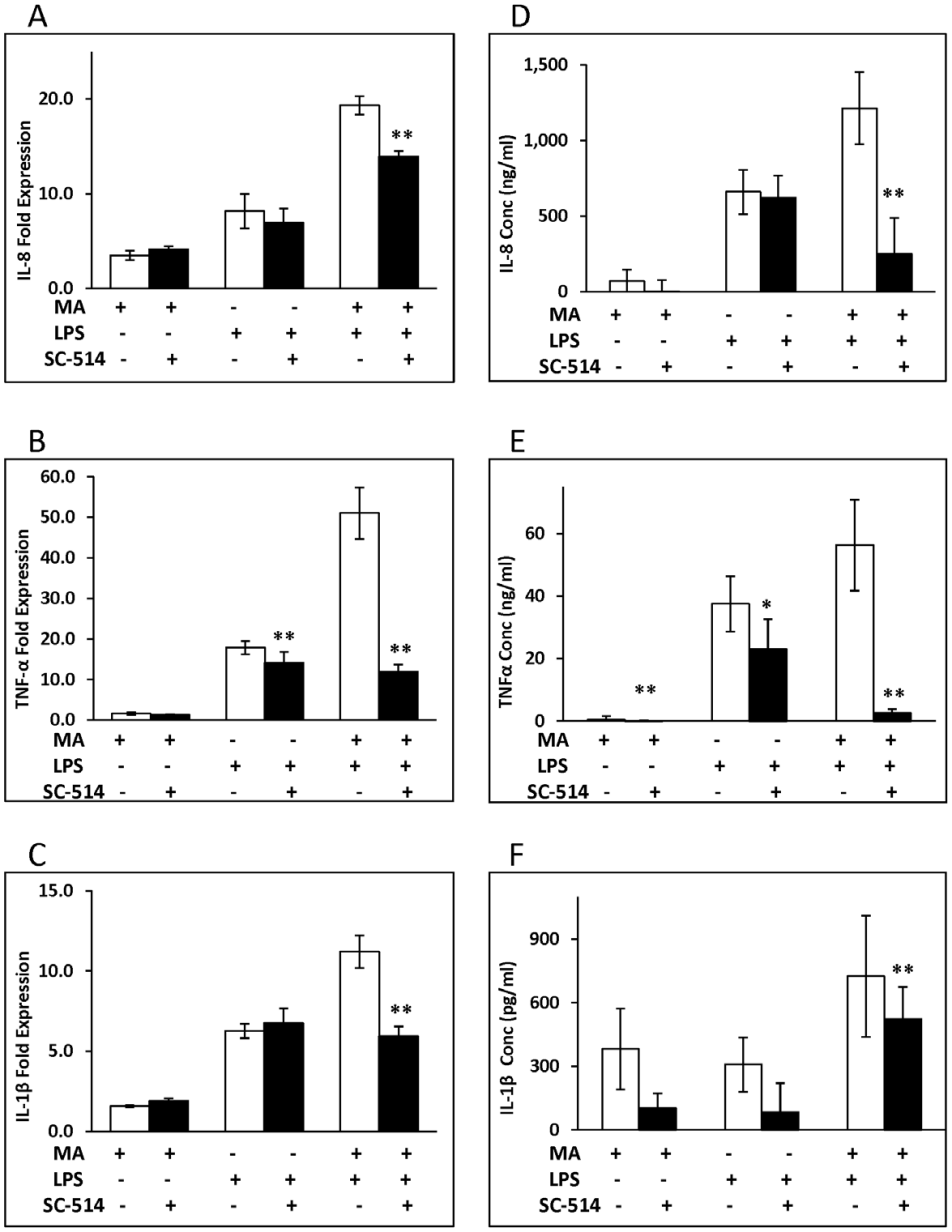
An inhibitor of NF-kB abrogates cytokine/chemokine expression induced by MA ± LPS. (A–C)1×10^6^ U937 monocytes were differentiated into macrophages with 100 nM PMA for 48 h. IKK2 inhibitor SC-514 was added 1 h before each MA exposure at 0, 24, 48 h. Cells were treated with LPS and the final dose of MA and then harvested after 3 h for RNA isolation and real time RT-PCR analysis. The mRNA expression levels were compared to untreated control. (D–F) For cytokine protein levels, cell supernatants were harvested 24 h after the last MA ± LPS treatment. The protein concentrations were normalized by subtracting values of control or inhibitor only. The means and SE values are from three independent experiments. *:p<0.05; **: p<0.01 comparing to MA/LPS/LPS+MA treatment as positive controls.

Preincubation of U937 macrophages with an inhibitor of IKK2 of the NF-κB pathway, SC-514, resulted in significant inhibition of TNF-α at the levels of both protein and mRNA (Fig. 3B and 3E). Although pretreatment of U937 macrophages with SC-514 prior to treatment with MA and LPS resulted in inhibition of IL-8 and IL-1β in terms of both protein or RNA, the repression of cytokine levels did not reach statistical significance (e.g. Fig. 3A, 3D, 3C, and 3F). However, SC-514 did cause significant inhibition of IL-8, TNF-α, and IL-1β protein and RNA levels that were induced by treatment with both MA and LPS (Fig. 3A–F).

### The MAPK and PI3-Akt pathways mediate the induction of cytokines in macrophages stimulated with MA and LPS

**Figure 4:**
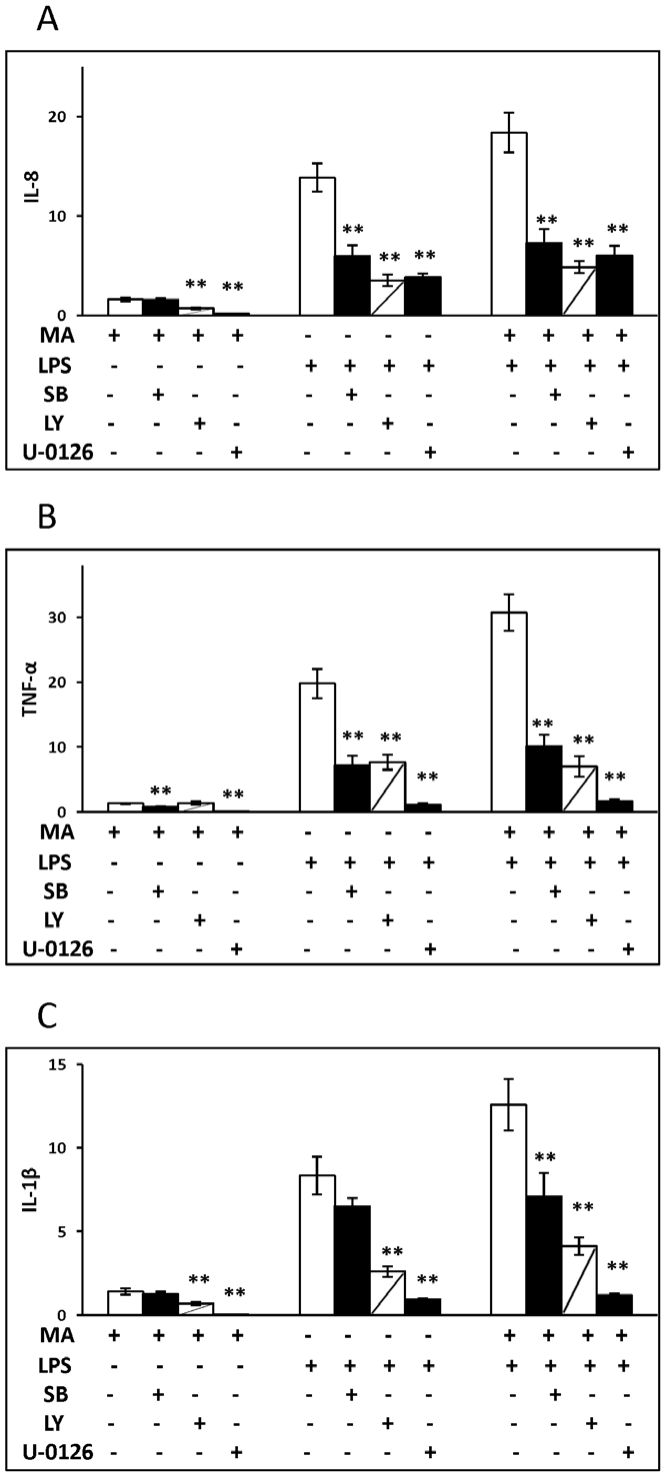
Inhibitors of MAPK or PI3-Akt abrogate cytokine/chemokine expression induced by MA ± LPS. 1×10^6^ U937 monocytes were differentiated into macrophages by treatment with 100 nM PMA for 48 h. p38 inhibitor SB203580, PI3K/Akt inhibitor LY294002 and ERK1/2 inhibitor U-0126 were added 1 h before MA exposure at 0, 24, 48 h. Cells were then challenged with LPS and the final dose of MA. After 3 h cells were harvested, RNA isolated and analyzed by real time RT-PCR. The means and SE values are from three independent experiments. *:p<0.05; **: p<0.01. Expression levels were compared to the identical treatment without inhibitor.

In order to determine the role of signaling pathways in the induction of inflammatory cytokines by MA and LPS, U937 macrophages were treated with inhibitors for p38 MAPK (SB203580), ERK1/2 (U-0126), or PI3/Akt (LY294002) starting at 1 h before each of the three daily treatments with MA. Treatment with the ERK1/2 inhibitor inhibited the induction of all cytokines by MA, LPS, or MA in combination with LPS (Fig. 4A, 4B, and 4C). Treatment with the inhibitor of p38 MAPK inhibited induction of TNF-α by MA, as well as the induction of TNF-α and IL-8 by LPS or LPS/MA and the induction of IL-1β by LPS/MA treatment (Fig. 4A, 4B and 4C). Treatment with the inhibitor of PI3/Akt resulted in the inhibition of the induction of IL-8 and IL-1β by MA, as well as the inhibition of the induction of IL-8, IL-1β and TNF-α by LPS or LPS/MA (Fig. 4A, 4B, 4C).

## Discussion

The induction of inflammation in macrophages by LPS has been extensively studied for the past three decades. Although there are still questions that remain to be resolved, it is clear that LPS/TLR4 signaling involves the NF-κB, MAPK and PI3-Akt pathways (reviewed in [16], [17], [18]). In contrast, very little is known about the effects of MA on macrophages and the signaling pathways that mediate these effects. Our results demonstrate that LPS-treatment of macrophages exposed to MA for 48–72 h result in the induction of IL-8, TNF-α and IL-1β to levels that were greater than were seen by treatment of macrophages with LPS alone. Although, as is to be expected, the actual fold-differences were different for RNA and protein, the expression profiles obtained at the level of RNA were virtually identical to the expression patterns observed at the level of protein. At the level of protein, MA alone induced significantly higher levels of TNF-α and IL-1β. For all three cytokines/chemokines examined, the level of protein induced by treatment with both MA and LPS was significantly greater than the additive effects of each agent alone. This suggests that, at least in some cases, MA and LPS may interact synergistically to increase cytokine or chemokine production. This has significant clinical implications for the effects of drugs of abuse on inflammation, particularly with respect to that caused by infectious agents. Definitive proof of synergy requires treatment with additional doses of these agents and these studies are currently in progress. The induction of increased levels of IL-1β by concomitant treatment with MA and LPS confirms a previous report that demonstrated a similar phenomenon in IL-1β production as measured by ELISA of cell culture supernatants [19].

The results described above led us to inquire as to the mechanisms responsible for the cytokine induction. LPS/TLR4 signaling pathways have been well described, but the signaling pathways that mediate the effects of MA in macrophages remain largely unexplored. However, it is known that macrophages express D1 and D2 dopamine receptors. Through the use of selective D1 or D2 inhibitors, it has been demonstrated that the effects of MA in macrophages are mediated through either D1 or D2 dopamine receptors [10], [11]. Signaling through the D1 and D2 receptors has been demonstrated to be mediated through a number of pathways, including the NF-κB pathway [24], [25], the MAPK pathways [24], [26], [27], and the PI3-Akt pathway [28], [29], [30], [31].

The first pathway we investigated was the NF-κB pathway. Through the use of cytoplasmic and nuclear extracts, we determined that treatment of U937 macrophages with MA for 48 h alters the nuclear∶cytoplasmic ratio of the p50 subunit of NF-κB. Cells treated with either LPS, or a combination of LPS and MA showed an even greater effect in terms of the increase in p50 nuclear∶cytoplasmic ratio. Although there was little difference between the nuclear∶cytoplasmic ratios observed between the cells treated with LPS and those treated with both LPS and MA, we postulate that this may be due to the ratio having reached its maximum. The role of the NF-κB pathway in mediating the induction of inflammatory cytokines in response to LPS and MA was confirmed using SC-514, an inhibitor of IKK2. Although the effects of SC-514 treatment on the induction of IL-8, TNF-α and IL-1β by MA at the level of RNA were slight, the inhibitory effect of SC-514 as shown by decreased cytokine production was more apparent at the protein level. It is worthwhile to note that while SC-514 inhibits IKK-2 (i.e. IKK-β), this kinase is thought to be dispensable for LPS-mediated activation of NF-κB in macrophages and monocyte-derived dendritic cells [32], [33]. In our system the activity of IKK-α (i.e. the non-canonical NF-κB pathway, reviewed in [34]), or activation of cytokine production by an alternative pathway (e.g. MAPK) may be the cause of the lack of efficacy of SC-514 in inhibiting cytokine production in LPS-stimulated macrophages. Furthermore, the effects of SC-514 were statistically significant for all cytokines in terms of induction by concomitant treatment with both LPS and MA, at both protein and RNA levels. Taken together with the changes produced by the different treatments on the nuclear-cytoplasmic distribution of p50, this confirms that the NF-κB pathway is a key mediator of the effects of MA on LPS-stimulated macrophages.

In addition to the NF-κB pathway, the MAPK signaling pathway has also been demonstrated to mediate D1 and D2 signaling [24], [26], [27], [35]. MA has been shown to affect phosphorylation of ERK and p38 MAPK in macrophages [10] and monocyte-derived dendritic cells [14]. We also determined the role of the PI3-Akt pathway, as this pathway has also been shown to modulate TLR4/LPS signaling [36] [37], as well as signaling through D1 and D2 [28], [29], [30], [31]. Our results demonstrated that p38MAPK and ERK1/2 are involved in mediating MA-induction of TNF-α. Our studies also show that MA-inductions of IL-8 and IL-1β are mediated by the PI3-Akt pathway and ERK1/2.

Taken together with the results discussed above, it is important to note that the use of any one of the signaling inhibitors, i.e. inhibitors for p38 MAPK, PI3-Akt, ERK1/2, or NF-κB, results in significant abrogation of MA-mediated increases of IL-8, IL-1β and TNF-α. This is most probably because the pathways that mediate MA-induced increases in these inflammatory cytokines are also involved in the induction of cytokines in response to LPS. We propose that it is through the utilization of these overlapping pathways that MA exerts its effects on LPS-stimulated macrophages. Furthermore, as we have noted, while the use of an inhibitor of IKK2 results in the inhibition of the LPS/MA cotreatments, the ERK1/2 inhibitor abrogated cytokine induction in response to the cotreatments with LPS and MA, as well as to the treatments with LPS and MA individually. This may suggest that ERK signaling is downstream of NF-κB signaling. A more definitive analysis of the sequence of induction of these pathways is currently in progress.

Although we have demonstrated that the NF-κB and MAPK signaling pathways play key roles in the induction of inflammatory cytokines by LPS and MA, the roles other signaling components and pathways involved in this phenomenon remain to be elucidated. For example, although dopamine receptors has been shown to be expressed in macrophages (10, 11), direct binding of MA to these receptors has not been demonstrated. In addition, while we have demonstrated that the 2 major signaling pathways in the induction of cytokines by LPS and MA, the effects of MA on the upstream components of the TLR4 signaling pathway (e.g. CD14, MyD88, TRAF6, etc.) have yet to be determined. It is quite possible that MA affects LPS signaling by affecting the expression or function of any of these components.

Our finding of the increased production of inflammatory cytokines in response to MA also has important implications in terms of the clinical effects of MA abuse. Numerous studies have demonstrated a strong linkage between MA abuse and HIV infection. MA abuse tends to increase risky sexual behaviors, and is thus a contributing factor to the acquisition of HIV infection [1], [2]. In the early stages of HIV infection the virus enters the CNS, most probably through trafficking of infected monocytes across the blood-brain barrier (BBB). In a trend that has been increasing in the past few years, the effects of HIV infection of the CNS, a syndrome known as HIV-associated neurocognitive disorder (HAND), have become more prevalent. Current thought is that one of the major factors responsible for HAND is increased neuroinflammation due to cytokine production induced by HIV-1 neurotoxins. A recent report has demonstrated that MA increases levels of matrix metalloproteinase 9 (MMP9) in LPS-treated macrophages [20]. Increased levels of this enzyme are associated with increased trafficking of infected monocytes across the BBB [38]. Taken together with our finding of MA-mediated induction of increased levels of inflammatory cytokines, this suggests that MA could operate on several levels to increase the prevalence of HAND in HIV-infected abusers of the drug. Thus, MA may not only increase the level of monocyte trafficking across the BBB, but it may also act to increase the production of inflammatory cytokines that are produced in microglia.

In summary, our results demonstrate that exposure to MA increases the levels of the inflammatory cytokines IL-8, IL-1β and TNF-α in LPS-treated macrophages. This is the first report that has demonstrated the induction of inflammatory cytokines by MA treatment of macrophages. The pathways that we have identified as being involved in MA-mediated induction of these cytokines are also key players in LPS-mediated inflammation. The exacerbation of cytokine production in response to both agents is likely the result of the utilization of overlapping signaling pathways activated by MA and LPS. This finding has important implications because of the potential of MA to exacerbate other inflammatory conditions that also modulate the signaling pathways identified in this study.
